# The Role of Pd-Pt Bimetallic Catalysts in Ethylene Detection by CMOS-MEMS Gas Sensor Dubbed GMOS

**DOI:** 10.3390/mi16060672

**Published:** 2025-05-31

**Authors:** Hanin Ashkar, Sara Stolyarova, Tanya Blank, Yael Nemirovsky

**Affiliations:** 1 Electrical and Computer Engineering Department, Technion—Israel Institute of Technology, Haifa 32000, Israel; haninashkar@campus.technion.ac.il (H.A.); ssstolya@g.technion.ac.il (S.S.); tblank@technion.ac.il (T.B.); 2 Todos Technologies Ltd., Kinneret 12, Airport City 7019900, Israel

**Keywords:** ethylene sensor, CMOS-SOI-MEMS, bimetallic catalyst, Pd-Pt, Pt

## Abstract

The importance and challenges of ethylene detection based on combustion-type low-cost commercial sensors for agricultural and industrial applications are well-established. This work summarizes the significant progress in ethylene detection based on an innovative Gas Metal Oxide Semiconductor (GMOS) sensor and a new catalytic composition of metallic nanoparticles. The paper presents a study on ethylene and ethanol sensing using a miniature catalytic sensor fabricated by Complementary Metal Oxide Semiconductor–Silicon-on-Insulator–Micro-Electro-Mechanical System (CMOS-SOI-MEMS) technology. The GMOS performance with bimetallic palladium–platinum (Pd-Pt) and monometallic palladium (Pd) and platinum (Pt) catalysts is compared. The synergetic effect of the Pd-Pt catalyst is observed, which is expressed in the shift of combustion reaction ignition to lower catalyst temperatures as well as increased sensitivity compared to monometallic components. The optimal catalysts and their temperature regimes for low and high ethylene concentrations are chosen, resulting in lower power consumption by the sensor.

## 1. Introduction and Objectives

The precise measurement of ethylene concentrations is extremely important for smart agriculture, the food supply chain, and the chemical industry. Currently, the detection of ethylene, a critical parameter in predicting fruit shelf life, is limited by the lack of suitable measurement systems capable of detecting ethylene variations in the several parts per million range and in real time [1]. Existing sensor technologies are not equipped to withstand the harsh conditions inside containers during transport, in storage rooms, or in industrial plants. Standard Volatile Organic Component (VOC) sensors and other ethylene sensors cross-react with the other gases present in produce supply chains, lack sensitivity, and fail to provide stable, long-term readings [1].

**Figure 1:**
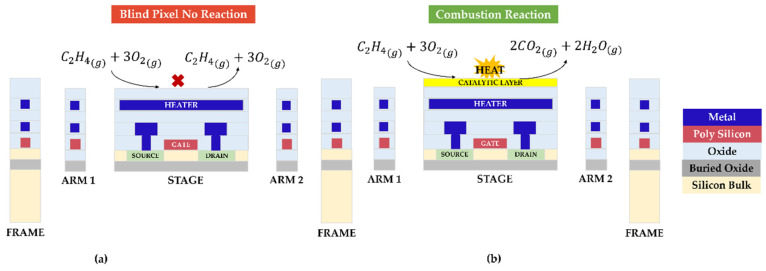
Schematic differential gas detection by the GMOS sensor. The “Blind” (**a**) and “Active” (**b**) pixels are heated with an embedded heating resistor to the ignition temperature of the analyte gas (ethylene in this illustration). The combustion reaction takes place on the “Active” pixel only, so the temperature of this pixel increases and modifies the current-voltage characteristics of the sensing transistor. In operation, the V_DS_ difference between “Blind” and “Active” transistors is measured. Therefore, the voltage response is proportional to ΔT_Reaction_.

In [2,3,4,5,6,7], we reported on a Complementary Metal Oxide Semiconductor–Silicon-on-Insulator–Micro-Electro-Mechanical System (CMOS-SOI-MEMS) catalytic gas sensor, dubbed the Gas Metal Oxide Semiconductor (GMOS), for the sensitive and selective detection of volatile organic compounds, briefly including ethylene. GMOS is a pellistor-like gas sensor fabricated using CMOS-SOI-MEMS technology. The sensing element is a suspended transistor equipped with an integrated heater and covered with a nanoparticle catalyst. When ethylene is present in the air, it triggers an exothermic combustion reaction on the catalytic layer, resulting in a change in the sensing transistor’s temperature and current-voltage characteristics, indicating the presence of the gas. By measuring the gas differentially between pixels with and without catalysts, the GMOS provides an assessment of the ethylene concentration based on the degree of temperature change caused by the chemical reaction (Figure 1).

To activate the chemical reaction, the pixel with the catalytic layer is heated by an embedded heating resistor made of tungsten. The catalyst temperature is controlled by the embedded heater voltage, which is adjusted based on the target gas and the type of catalyst. Each gas–catalyst pair has a specific relationship between the GMOS signal and the heater voltage. By analyzing this relationship, optimal operating points can be selected. Measuring the GMOS signal at two or more different heater voltages (temperatures) enables the selective detection of different gases.

Our previous paper covered different issues relating to gas sensing, such as the following: (i) enhanced sensitivity due to the use of a nanoparticle catalyst [3]; (ii) a selective approach using a temperature pattern by measuring the GMOS response as a function of the catalyst temperature for each gas of interest and data processing using Machine Learning (ML) Artificial Intelligence (AI) algorithms [5]; (iii) power consumption optimization using computer simulation [4]; improvements in sensing stability over time [7]; humidity effects [6].

The catalyst plays a critical role in addressing all aspects of gas sensing performance. The activity of the catalysts depends on both their chemical compositions and preparation method, as these factors can significantly affect the surface structure of active metals. In the past, for the GMOS catalytic gas sensor, we used monometallic catalysts such as palladium (Pd) and platinum (Pt). We started by using a Pd catalyst for measuring relatively high concentrations of 500 parts per million (ppm) ethanol and acetone [2]. In our later papers, we applied Pt as a catalytic layer for sensing a wide concentration range of 1–700 ppm of ethanol [3,4,5,6], as well as Pt and TiO_2_ catalysts for 1–100 ppm of ethylene [7].

Recently, it was reported that bimetallic catalysts show remarkably well-modified catalytic properties compared to their monometallic counterparts and influence the selectivity and stability of the catalytic layer [8,9,10,11,12,13]. The effect of bimetallic catalysts has not been shown for ethylene so far. Taking this into account, in the present work, we included a Pd-Pt catalyst and performed a comparative study of Pt, Pd, and Pd-Pt catalysts for the GMOS sensing of ethylene and ethanol. The sensitivity–temperature dependence, revealing the ignition temperature, was measured for these catalysts. Assessment of the temperature dependence is important for the proper choice of operating temperature for the sensor. It helps find an optimal operating temperature for reaching high sensitivity along with low power consumption. Moreover, the ratio of signal values at different temperatures creates a pattern recognition for the selective sensing of a specific gas.

The primary objectives of this study are as follows:

(1) Choosing optimal catalyst and operation regimes for the GMOS sensing of ethylene at low and high concentrations.
(2) Comparative analysis of Pt, Pd, and Pd-Pt catalysts for the GMOS sensing of ethylene and ethanol.

## 2. Experimental

### 2.1. Measurement of GMOS Response

**Figure 2:**
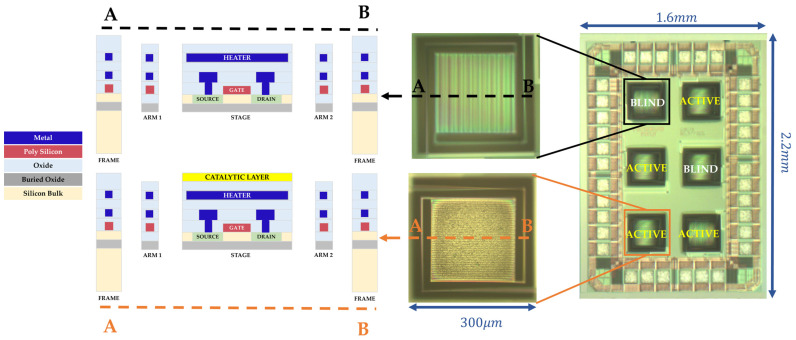
A 1.6 × 2.2 mm GMOS sensor die featuring active and blind pixels, as captured in an optical microscope. The left side shows a cross-sectional view of both pixel types, which differ by the presence of a catalytic layer in the active pixel.

**Figure 3:**
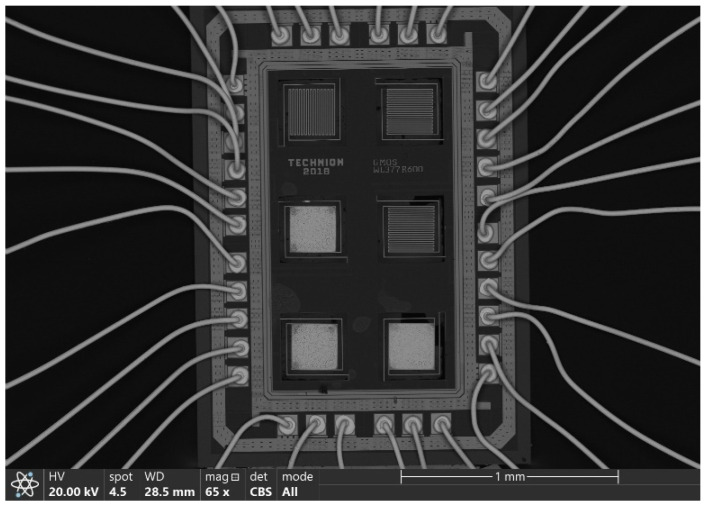
SEM image of the fabricated GMOS device, showing both free pixels and pixels covered with a Pd-Pt catalyst.

The GMOS die, with an overall area of 1.6 × 2.2 mm, contains six identical 300 × 300 µm micromachined pixels (Figure 2). Four of them are active pixels, covered with either identical or different catalytic layers, and the remaining two, referred to as reference or “blind” pixels, are not coated with catalysts and are used for differential temperature measurements. The voltage signal caused by a temperature change due to gas combustion on each active pixel (with a catalyst) can be separately measured relative to one of the blind pixels. A SEM image of the GMOS sensor die is shown in Figure 3.

**Figure 4:**
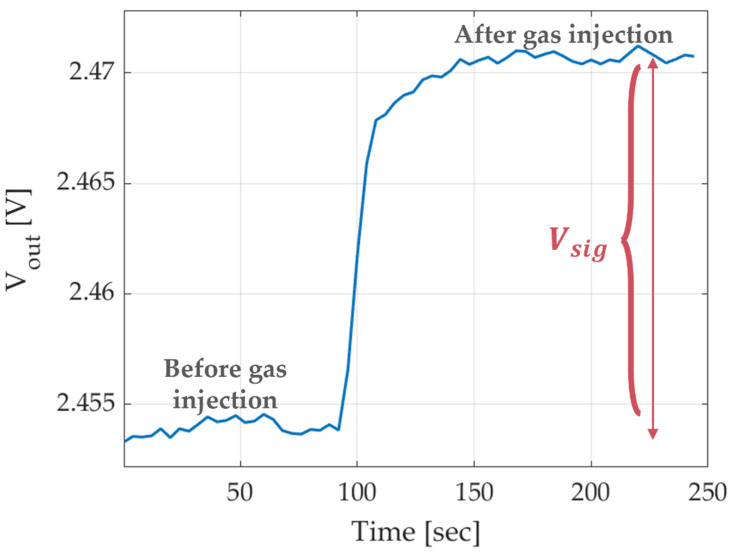
Signal response of the GMOS device over time during gas injection, illustrating how *V_sig_* is calculated as the difference between the signal after and before gas exposure.

The output voltage *V_out_* of the GMOS device is the difference in drain-source voltage between the two transistors: one in the pixel with the catalytic layer, and the other in a pixel without it. Both pixels possess nearly thermal properties, and the differential approach effectively eliminates the impact of transistor self-heating and variations in ambient temperature on GMOS output. So, changes in *V_out_* are correlated with the temperature increase resulting from the heat that occurs specifically due to gas exposure in the catalytic layer [2]. The *V_sig_* signal represents the change in *V_out_* that occurs specifically due to gas exposure. Figure 4 illustrates the typical signal response of the GMOS device over time during gas injection.

*V_sig_* is proportional to the pixel temperature change caused by the chemical reaction [3], as described in Equation (1).

(1) $$V_{sig}=\left(\frac{dV_{DS}}{dT}\right)\cdot \Delta T_{Reaction}=\left(\frac{dV_{DS}}{dT}\right)\cdot \left(\frac{P_{Reaction}}{G_{TH}}\right)$$

*P_Reaction_* is the chemical reaction power, and it is given by the following:

(2) $$P_{Reaction}=Flux\cdot Area\cdot \left(\frac{\Delta H_{C}}{N_{A}}\right)$$

For the 1*^st^* order reaction and steady state

(3) $$Flux=C_{Gas}\cdot {\left(\frac{1}{k_{S}}+\frac{\delta }{D}\right)}^{-1}\left[\frac{ppm}{m^{2}\cdot s}\right]$$

So, *V_sig_* can be expressed as follows:

(4) $$V_{sig}=\left(\frac{dV_{DS}}{dT}\right)\cdot Area\cdot C_{Gas}\cdot {\left(\frac{1}{k_{S}}+\frac{\delta }{D}\right)}^{-1}\cdot \left(\frac{\Delta H_{C}}{N_{A}\cdot G_{TH}}\right)$$

where V_DS_ is the drain-source voltage of the transistor, V; T is the transistor temperature, K; $k_{S}$ is the reaction rate, m/s $\left(k_{S}=Aexp(-E_{a}/KT\right)$; C_Gas_ is the gas concentration in air, molecules/m^3^; D is the gas diffusion constant, m^2^/s; δ is the stagnant film thickness, m; G_TH_ is the thermal conductance, W/K; $\Delta H_{C}$ is the combustion enthalpy, Joule/mole; N_A_ is the Avogadro number, 1/mole.

The enthalpy of the ethylene combustion reaction presented below is −1410.97 ± 0.30 kJ/mol [14].

(5) $$C_{2}H_{4}+3O_{2}\to 2CO_{2}+2H_{2}O$$

For a given catalyst, the GMOS response depends on both the gas concentration in the environment and the pixel catalyst temperature. In the range of relatively low gas concentrations, the higher the gas concentration, the more molecules available to produce a reaction on the catalytic surface and the stronger the GMOS response is supposed to be. The dependence on the temperature is more complicated because there are two temperature regions: reaction-controlled and diffusion-controlled. In the reaction-controlled region, the response increases exponentially with the temperature, while in the diffusion-controlled regime, the signal is nearly independent of the temperature [15]. The catalyst quality is estimated according to the ignition temperature, which is determined at the transition to the diffusion-controlled region, and the GMOS sensitivity (S [V/ppm]) at given pixel temperatures:

(6) $$S=\frac{V_{sig}}{C_{gas}}$$

where S is the sensitivity [V/ppm], V_sig_ is the output voltage change [V], and C_gas_ is the gas concentration in the chamber.

The ideal catalyst for catalytic combustion should exhibit excellent low-temperature activity as well as chemical and thermal stability.

### 2.2. Preparation of Catalytic Layers

For the platinum catalytic layer, 10% Pt nanoparticle ink from Fraunhofer Institute, Dresden [16], was used, representing Pt nanoparticles in a solvent. The palladium (Pd) catalytic layer was prepared using ink from Heraeus Holding Gmb [17] containing dissolved Pd compounds, which were transformed into Pd nanoparticles on the heated pixel surface after deposition. The Pt ink was diluted with thinner at a 1:2 ratio, while the Pd ink was applied without any dilution.

Catalyst deposition was performed manually using a fine-tipped brush, allowing for controlled and localized application. Following deposition, the sensor was placed on a hot plate and heated at approximately 200 °C for 30 min to facilitate solvent evaporation, drying, and catalyst solidification.

The bimetallic Pd-Pt catalyst represented a two-layer structure on the GMOS pixel. The first layer was made from a Pd solution, which was heated to form a solid layer before the Pt layer was deposited. Then, the bilayer structure was heated together for solidification.

Note that while sophisticated synthesis methods for Pd-Pt core-shell and alloy nanostructures have been reported in the literature [11,12,13], the focus of this work is on cost-effective and simple technology for gas sensing. Therefore, the decision was made to use commercially available Pd (Heraeus) and Pt (Fraunhofer) catalysts for the GMOS sensor.

### 2.3. Gas Injection Procedure

**Figure 5:**
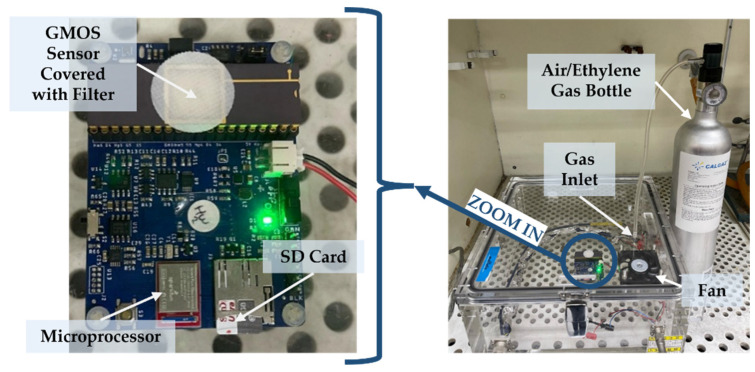
Experimental setup including a chamber, gas supply, and evaluation board with the GMOS sensor, computer-controlled via Bluetooth.

Ethylene was supplied from a calibrated gas cylinder containing an air/ethylene mixture (0.02% or 200 ppm ethylene, ±2% accuracy), purchased from CALGAZ [18]. A flow regulator was employed to precisely control the volume of ethylene injected into the system. The experimental setup is presented in Figure 5.

The concentration of ethylene gas in the chamber was calculated using Equation (7), which is based on a fundamental mass balance [19], where the injected gas volume is expressed as the product of the flow rate and the injection time:

(7) $$C_{ch}=\frac{C_{0}\times f\times t}{V_{ch}+f\times t}$$

where *C_ch_* is the ethylene concentration in the chamber; *C*_0_ is the ethylene concentration in the bottle; *f* is the gas mixture flow; *V_ch_* is the air volume in the chamber; and *t* is the introduction time.

Ethanol was introduced in liquid form using a calibrated syringe to deposit a precise volume onto a surface inside the sealed chamber. A built-in fan was then run for 10–15 s to ensure complete evaporation and uniform vapor distribution. The ethanol used was absolute, dehydrated (99.9% purity), purchased from Biolab Ltd., Jerusalem, Israel (catalog number 052551) [20].

For ethanol, the concentration was calculated using the ideal gas law, following the approach described in [21]. However, the concentration was expressed in ppm instead of molar units. To obtain an ethanol concentration of 1 ppm, the required volume in liters can be calculated using the following equation:

(8) $$V_{ethanol,1ppm}=\frac{P_{ch}\cdot V_{ch}\cdot {M_{w}}_{ethanol}}{10^{6}\cdot R\cdot T\cdot \rho_{ethanol}}$$

where P_ch_ is the atmospheric pressure inside the chamber (1 atm), V_ch_ is the chamber volume (6 L), R is the gas constant (8.314 J/mol·K), T is temperature (300 K), ${M_{w}}_{ethanol}$ is the ethanol molecular mass (46.07 g/mol), and $\rho_{ethanol}$ is ethanol density (0.78 × 10^−3^ gr/L).

Thus, to get X ppm of ethanol, we need to add X times the volume we calculated for 1 ppm (V_ethanol,1ppm_).

## 3. Results and Discussion

### 3.1. Morphology and Composition of Catalytic Layers

**Figure 6:**
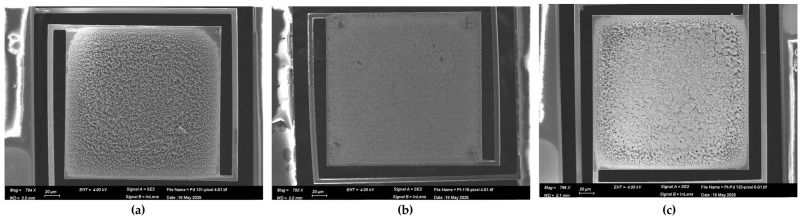
SEM general view of pixels covered with catalytic layers, captured at a magnification of 795×. (**a**) Pd catalyst, (**b**) Pt catalyst, (**c**) Pd-Pt catalyst.

The nanostructure morphology of the Pd, Pt, and Pd-Pt catalytic layers was investigated using a high-resolution scanning electron microscope (HR–SEM). Specifically, a Zeiss Ultra Plus field emission SEM was employed at a primary electron energy of 4 keV, utilizing both Everhart–Thornley and in-lens secondary electron detectors to achieve detailed surface imaging with high topographical sensitivity. Elemental composition was characterized by another SEM system (Prisma) equipped with energy-dispersive X-ray analysis (EDX). This system operated at 20 and 30 keV and included an Everhart–Thornley secondary electron detector along with a solid-state backscattered electron detector functioning in both Z-contrast and topographical modes. The thickness of the catalytic layers was estimated using Focused Ion Beam (FIB) milling with a Helios G3 Dual Beam (Thermo Fisher Scientific—TFS, Waltham, MA, USA). A representative view of the catalyst-coated pixels is presented in Figure 6.

**Figure 7:**
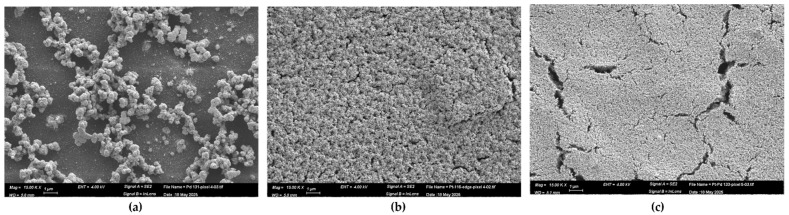
SEM images of catalytic layers, captured at a magnification of 15,000×: (**a**) Pd catalyst, (**b**) Pt catalyst, (**c**) Pd-Pt catalyst.

In Figure 6a, the Pd layer appears discontinuous compared to the more uniform Pt layer (Figure 6b). In the Pd-Pt layer (Figure 7c), the top Pt layer exhibits numerous cracks over the underlying Pd layer.

Figure 7a shows that the Pd catalytic layer exhibits vertical growth, forming agglomerated nanoparticles that stack upward rather than uniformly covering the pixel surface. In contrast, the Pt catalytic layer (Figure 7b) is composed of densely packed nanoparticles that form a continuous and uniform layer with minimal voids. This morphology provides a high surface area and a large number of active sites, enhancing gas adsorption and catalytic reaction efficiency. For the Pd-Pt bilayer (Figure 7c), as previously described, the structure consists of a Pd base layer followed by a Pt top layer. The SEM image reveals numerous cracks in the Pt layer. These cracks facilitate gas diffusion through the Pt layer, allowing interaction at the Pd-Pt interface and thereby potentially enhancing catalytic activity.

**Figure 8:**
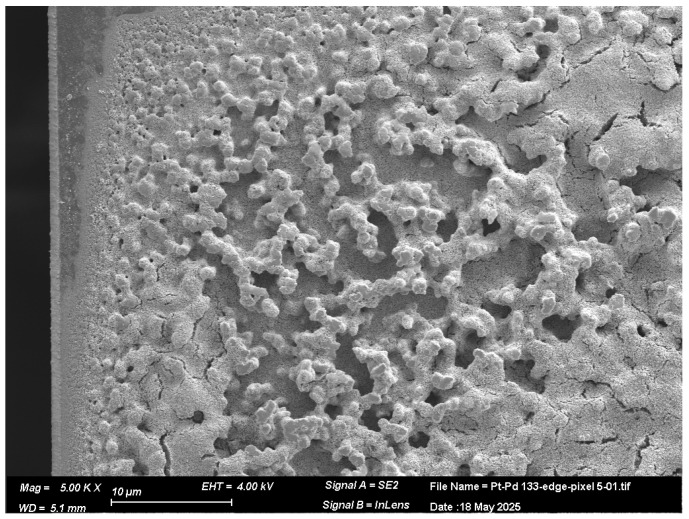
SEM image of the edge of a pixel coated with a Pd-Pt catalytic layer, captured at a magnification of 5000×.

Figure 8 provides a closer view of the edge of the pixel and the Pd-Pt bilayer, highlighting the morphological interaction between the two layers. The image reveals how the Pt layer conforms to the underlying vertically grown Pd nanostructures, resulting in a textured and uneven surface. The Pt deposition appears to partially fill the gaps between the Pd features but does not fully level the surface, leading to pronounced topographical variations. This complex 3D morphology likely increases the effective surface area and may influence gas transport dynamics across the catalyst surface, contributing to enhanced catalytic performance under certain conditions.

**Figure 9:**
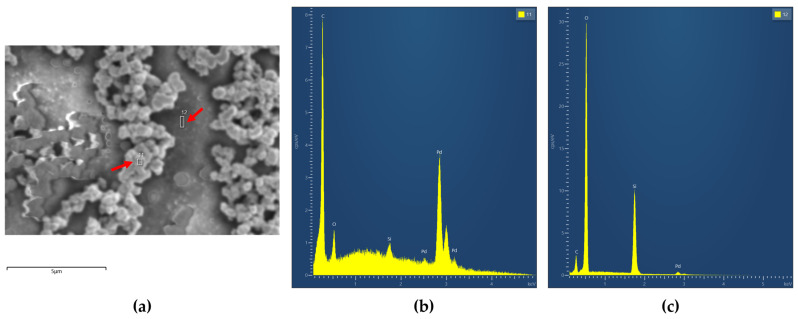
SEM—EDX spectrum of the Pd catalytic layer; (**a**) SEM image, (**b**) EDX spectrum from position 11, (**c**) EDX spectrum from position 12.

Figure 9a shows the SEM image of the Pd layer with marked points where EDX analysis was performed. The corresponding spectrum is presented in Figure 9b,c. The spectrum in Figure 9b confirms the presence of palladium, indicating successful deposition of the Pd layer at that location. In contrast, Figure 9c, taken from a different point on the same pixel, shows dominant silicon (Si), oxygen (O), and tungsten (W) signals, which originate from the substrate and the heater, and a significantly reduced palladium signal, suggesting that the Pd layer is very thin and may not cover the entire pixel surface.

**Figure 10:**
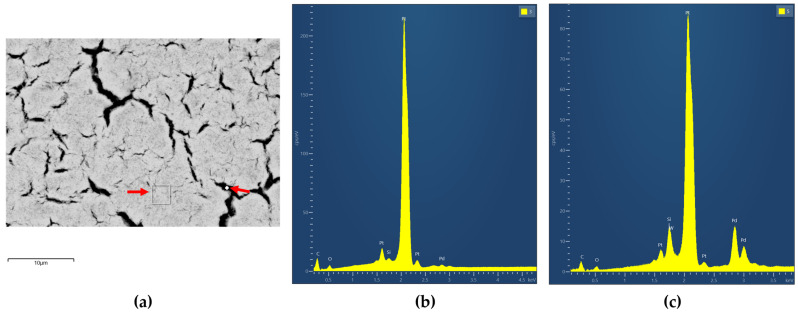
SEM—EDX spectrum of the Pd-Pt catalytic layer as captured at the center of the pixel; (**a**) SEM image, (**b**) EDX spectrum from position 3, (**c**) EDX spectrum from position 5.

Figure 10b,c displays EDX spectra acquired from distinct regions of the Pd-Pt bilayer, corresponding to the SEM image of the central area of the pixel (Figure 10a), where the bilayer appears relatively uniform yet exhibits surface cracks. In Figure 10b, which represents an area without visible cracks, the EDX spectrum suggests a predominantly platinum composition. In contrast, Figure 10c, taken from a cracked region, reveals the presence of both palladium and platinum. This observation supports the existence of an interface between the two metal layers. Such an interfacial structure may enhance the effective surface area and influence gas transport properties across the catalytic surface, as previously discussed.

**Figure 11:**
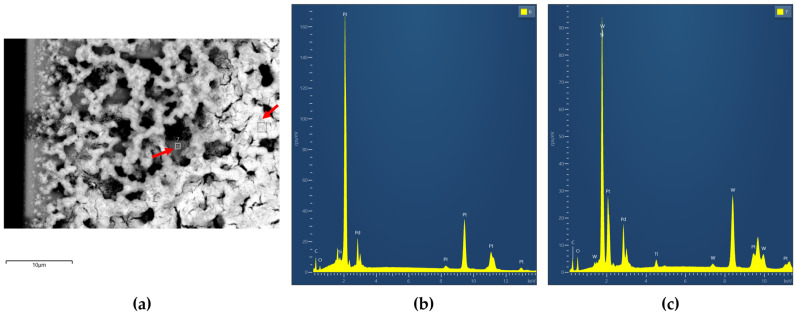
SEM—EDX spectrum of the Pd-Pt catalytic layer as captured at the edge of the pixel; (**a**) SEM image, (**b**) EDX spectrum from position 6, (**c**) EDX spectrum from position 7.

Figure 11b,c shows EDX spectra from different locations along the edge of the Pd-Pt-coated pixel, corresponding to the SEM image in Figure 11a. Both spectra confirm the presence of palladium and platinum.

**Figure 12:**
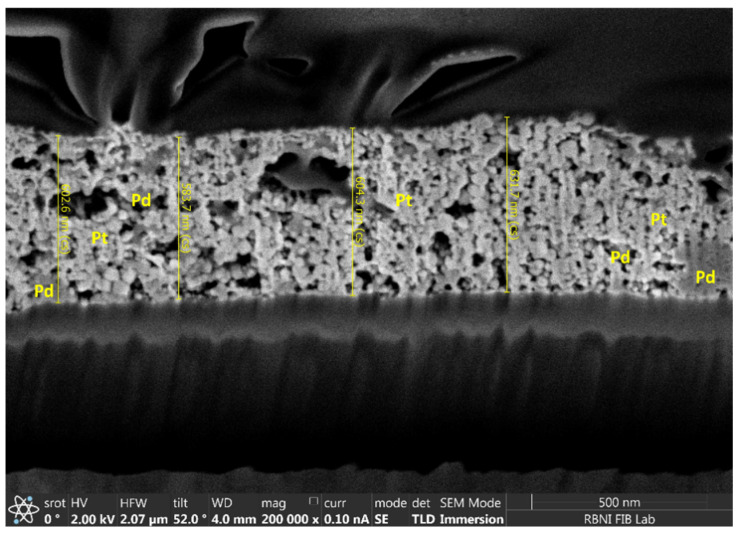
FIB image of the Pd-Pt catalytic layer.

To determine the thickness of the Pd-Pt and Pd catalytic layers, FIB analysis was performed on the Pd-Pt catalytic layer. As shown in Figure 12, Pd appears as darker regions compared to Pt. This contrast is due to the difference in atomic numbers. Pt has a higher atomic number than Pd, resulting in a greater yield of backscattered electrons and thus appearing brighter in the image. As described earlier, Pd tends to grow vertically rather than laterally, forming agglomerated nanoparticles. The overall thickness of the Pd-Pt catalytic layer was measured to be around 0.6 µm.

**Figure 13:**
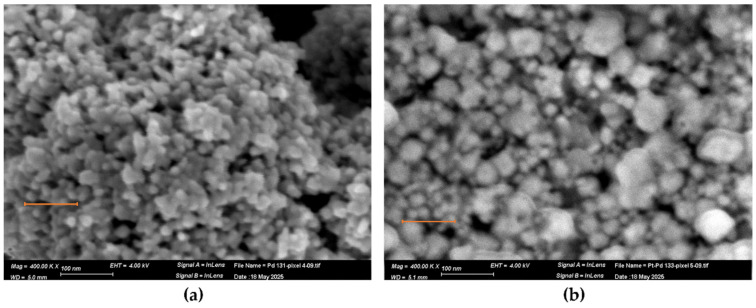
SEM images of (**a**) the Pd catalytic layer, (**b**) the Pd-Pt catalytic layer, as captured at a magnification of 400,000×.

According to Figure 13, both the Pd and Pd-Pt nanoparticles exhibit an average size of approximately 10–20 nm.

### 3.2. Sensing of Ethylene and Ethanol with Different Catalytic Layers

#### 3.2.1. GMOS Response Measurements

**Figure 14:**
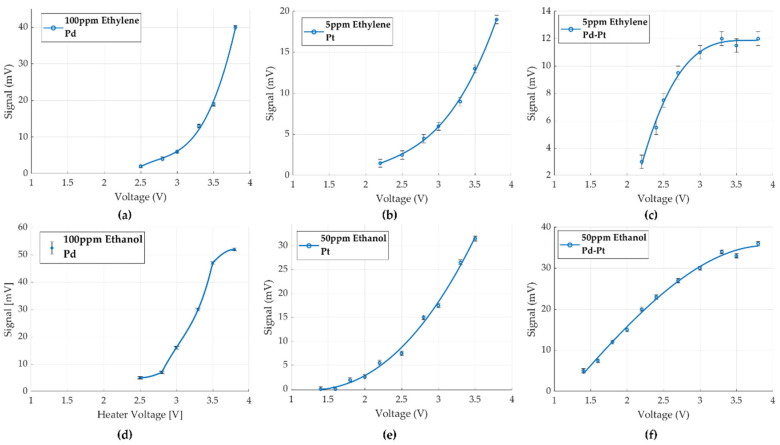
GMOS sensing of ethylene and ethanol with different catalysts as a function of heater voltage: (**a**) 100 ppm ethylene signal on the Pd catalyst, (**b**) 5 ppm ethylene signal on the Pt catalyst, (**c**) 5 ppm ethylene on the Pd-Pt catalyst, (**d**) 100 ppm ethanol signal on the Pd catalyst, (**e**) 50 ppm ethanol signal on the Pt catalyst, (**f**) 50 ppm ethanol on the Pd-Pt catalyst.

**Figure 15:**
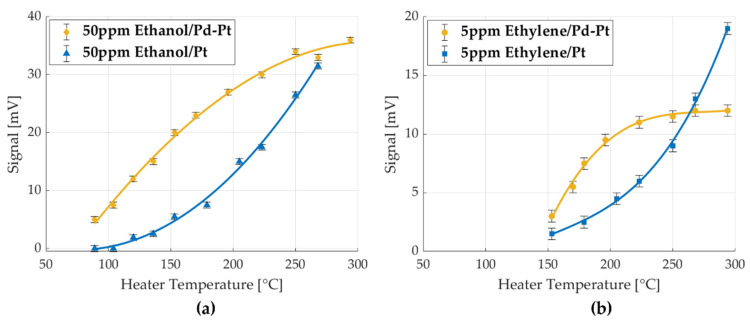
Temperature curves of the GMOS response to ethylene and ethanol with Pt and Pd-Pt catalysts. (**a**) 5 ppm ethylene signal on the Pd-Pt and Pt catalysts as a function of heater temperature, (**b**) 50 ppm ethanol signal on the Pd-Pt and Pt catalysts as a function of heater temperature.

The GMOS sensor’s response to ethylene and ethanol, measured at different heater voltages for several catalysts, is shown in Figure 14 and Figure 15. The catalyst temperatures were calculated using ANSYS Fluent 2022 R1 simulations based on the applied heater voltage, as detailed in Table 1 [6].

Figure 14a–c presents the comparative sensing behavior of the Pd, Pt, and Pd-Pt catalysts during ethylene injection, with signal variations confined within ±0.5 mV. Experiments conducted with Pd as the sole catalyst indicated that it does not respond to low ethylene concentrations. No sensing signal was observed unless the ethylene concentration in the chamber reached at least 100 ppm. Only after reaching this threshold and adding an additional 100 ppm did we measure an obvious response (Figure 14a). This result demonstrates that Pd is not sensitive to low ethylene levels and is therefore unsuitable for detecting low concentrations.

The plateau typically associated with the diffusion-controlled regime was not observed within the measured temperature range for Pt. Although the response with the Pt catalyst at the highest heater voltage (3.8 V) was stronger than that for Pd-Pt, at lower voltages, the response was superior for Pd-Pt.

Figure 14d–f presents the comparative sensing behavior of the Pd, Pt, and Pd-Pt catalysts during ethanol detection under the same experimental conditions, with signal variations confined within ±0.5 mV. A similar trend for ethanol was observed: Pd alone exhibited poor sensitivity, especially at low ethanol concentrations, Pt showed improved performance and a higher signal, and the Pd-Pt combination provided the best sensitivity, especially at lower operating voltages.

The Pd-Pt catalyst displayed an earlier onset of sensing and a stronger response at lower voltages than Pt, confirming that adding Pd also facilitates a reduction in the ignition temperature for ethanol sensing. These results are consistent with the behavior observed for ethylene and point to a synergistic catalytic effect between Pd and Pt, which enhances surface reactivity and lowers the activation energy for gas oxidation reactions.

Figure 15a,b presents a comparison of the GMOS sensor signal for ethylene and ethanol, respectively, using Pt and Pd-Pt catalysts across a range of heater temperatures with an error margin of ±0.5 mV. In both cases, the Pd-Pt catalyst exhibited superior performance, attributable to two key factors: (1) a shift in the diffusion-controlled regime to lower temperatures, indicating earlier onset of the temperature-independent response region, and (2) an increase in sensitivity, reflected in higher signal amplitude at comparable gas concentrations.

The earlier transition to the diffusion-controlled regime is particularly advantageous, as it improves signal stability by reducing dependence on heater voltage and allows for effective operation at lower temperatures. Lower operating temperatures, in turn, reduce power consumption and contribute to enhanced energy efficiency. The combination of these benefits makes the Pd-Pt catalyst more suitable for low-temperature, low-concentration gas sensing applications.

#### 3.2.2. Power Savings

The operating temperature directly influences the power consumption of the GMOS sensor. As demonstrated in the results, the use of a Pd-Pt catalyst enables sensing at lower operating temperatures, thereby reducing the overall power requirement.

The GMOS total power consumption includes both the transistor and the embedded heater. However, since the transistor operates in the subthreshold region, its power consumption is negligible [4]. The main contribution comes from the heater, whose power is calculated using Ohm’s law:

(9) $$P_{heater}=\frac{V_{heater}^{2}}{R}$$

where R is the heater resistance [Ω].

With the incorporation of the Pd-Pt catalyst, the ignition temperature of the target gas is reduced, enabling a lower operating voltage for ethylene sensing, from about 3.8 V using Pt to 3.2 V with Pd-Pt. At these voltages, the corresponding heater resistances are approximately 1080 Ω and 1000 Ω, respectively, resulting in a power reduction from 13.37 mW to 10.24 mW, a 23.41% decrease.

This improved sensing performance is also supported by the energy-efficient design of the GMOS sensor. The sensor is built on a suspended micromachined membrane with a very low thermal mass, allowing a fast thermal response and low energy requirements [4].

Furthermore, to minimize energy consumption without compromising sensing performance, the sensor can operate in pulsed mode, where the heater is periodically activated for a half duty cycle. This pulsed heating strategy maintains catalytic activity while substantially lowering the average power consumption, making it especially advantageous for battery-operated or energy-constrained applications [4].

#### 3.2.3. Comparison of Sensitivity Measurements

The comparison of GMOS sensitivities to ethylene and ethanol at two heater voltages of 3.5 V (268 °C) and 3.8 V (294 °C) with three catalysts is presented in Table 2. The results indicate that the GMOS sensitivity to ethylene on Pt and Pd-Pt is several times higher than that for ethanol. This observation, along with the differences in temperature dependences, creates an opportunity for selective ethylene sensing in the presence of ethanol.

**Figure 16:**
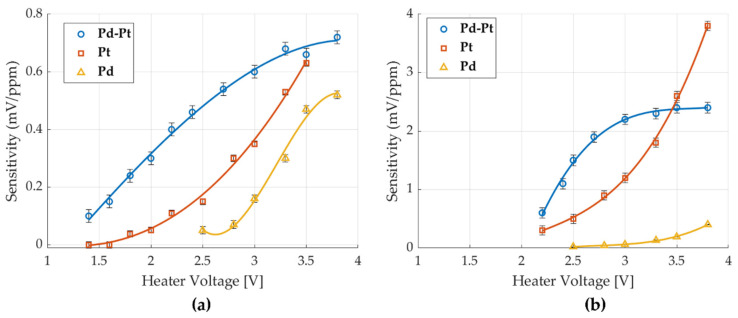
Sensitivity of different catalysts normalized to 1 ppm: (**a**) ethanol and (**b**) ethylene.

The sensitivity of the different catalysts to ethanol and ethylene is presented in Figure 16a,b, respectively. Sensitivity was calculated by dividing the measured voltage response (in mV) by the corresponding gas concentration (in ppm). For the Pd catalyst, measurements for both ethanol and ethylene were normalized using a concentration of 100 ppm. For the Pt and Pd-Pt catalysts, the sensitivity to ethylene was normalized using a concentration of 5 ppm, while for ethanol, it was normalized by 50 ppm.

The results show that the Pd catalyst exhibits the lowest sensitivity to both ethanol and ethylene, which is consistent with its limited response at low concentrations. For the Pt catalyst, a similar trend is observed for both gases—the sensitivity increases with heater voltage, and although not definitively exponential, the trend appears to follow a steep upward pattern at higher voltages. In contrast, the Pd-Pt catalyst displays a different behavior, where sensitivity increases with voltage up to a certain point, after which it stabilizes.

At lower heater voltages, below approximately 3.5 V (equivalent to ~268 °C), the Pd-Pt catalyst exhibits higher sensitivity than Pt and Pd for both ethanol and ethylene. However, at higher heater voltages/temperatures, the Pt catalyst demonstrates the highest sensitivity among the three.

#### 3.2.4. Activation Energy Analysis

**Figure 17:**
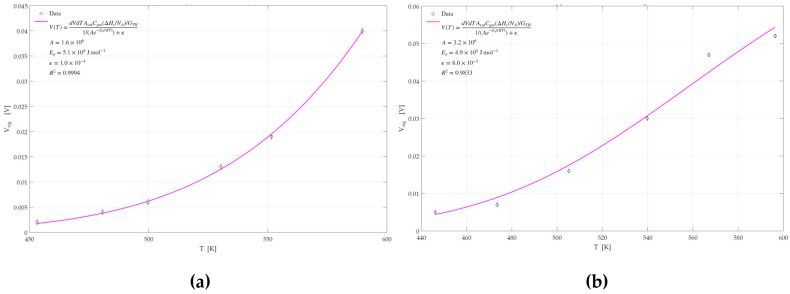
GMOS sensing of ethylene and ethanol on the Pd catalyst as a function of heater temperature: (**a**) 100 ppm ethylene signal on the Pd catalyst, (**b**) 100 ppm ethanol signal on the Pd catalyst.

**Figure 18:**
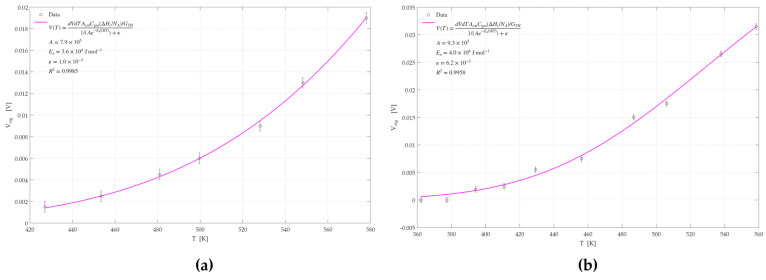
GMOS sensing of ethylene and ethanol on the Pt catalyst as a function of heater temperature: (**a**) 5 ppm ethylene signal on the Pt catalyst, (**b**) 50 ppm ethanol signal on the Pt catalyst.

**Figure 19:**
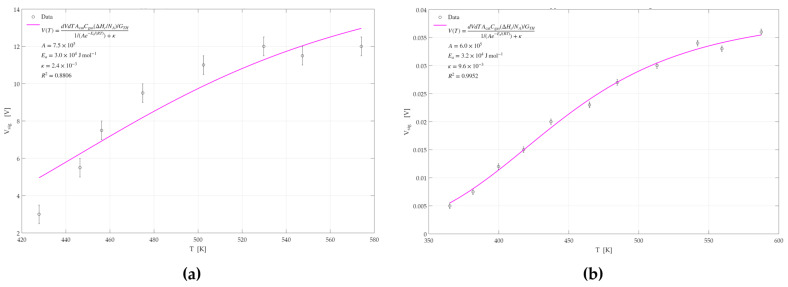
GMOS sensing of ethylene and ethanol on the Pd-Pt catalyst as a function of heater temperature: (**a**) 5 ppm ethylene signal on the Pd-Pt catalyst, (**b**) 50 ppm ethanol signal on the Pd-Pt catalyst.

The GMOS response measurements demonstrated superior sensitivity to ethylene and ethanol when using a Pd-Pt catalyst compared to the responses obtained with individual Pd or Pt catalysts. To further investigate whether this enhancement arises from kinetic effects or the morphological characteristics of the catalyst, an activation energy analysis was conducted. Simulations were performed based on Equation (4), allowing for the extraction of activation energies for each catalyst. This analysis was carried out for both ethylene and ethanol. The simulation results, generated using MATLAB 2022, are shown in Figure 17, Figure 18 and Figure 19. A summary of the calculated activation energies, the corresponding correlation coefficients for each of the six datasets, and the associated errors in the activation energy estimation are presented in Table 3.

From the results obtained, it can be observed that the Pd catalyst exhibits the highest activation energy for both ethylene and ethanol sensing, followed by the Pt catalyst. The Pd-Pt catalyst shows the lowest activation energy among the three. These findings indicate that the Pd-Pt catalyst facilitates the reaction more effectively, resulting in superior sensing performance and signal response compared to the other two catalysts.

For all six graphs, high R^2^ values were obtained, indicating a good fit and reliable extraction of activation energies. The exception is the R^2^ value obtained for the Pd-Pt catalyst for ethylene, which was relatively low (0.8805), suggesting that the corresponding activation energy may be less accurate. This can be attributed to the morphological characteristics of the Pd-Pt layer. SEM imaging and structural characterization revealed that this layer has observable regions with cracks and morphological variation. These variations suggest the presence of more and less active sites across the surface, which may affect the uniformity of the sensor’s response and warrant further depth analysis.

#### 3.2.5. Results Discussion

Based on morphological and structural characterization, activation energy, and sensitivity analysis, the Pd-Pt catalyst demonstrated superior performance compared to Pd and Pt alone. This enhanced performance is attributed to the synergistic effect of morphology and reduced activation energy. The distinct topography and surface morphology of the Pd-Pt layer appear to promote improved sensor activity, particularly in the detection of low concentrations of ethylene and ethanol. Additionally, the improved catalytic behavior is linked to the lower activation energy observed for the combustion of these gases.

The analysis also revealed that the Pd layer is not continuous but composed of vertically oriented structures (Section 3.1). These vertical features likely suffer from poor thermal contact with the underlying substrate, potentially leading to locally reduced temperatures. This thermal inefficiency may hinder catalytic reactions and explain both the reduced sensitivity and the higher observed activation energy. Furthermore, the limited effective surface area, critical to sensing performance due to its direct relationship with signal strength (Equation (4)), may further constrain sensor output.

Concerning catalytic mechanisms, the active phase of Pd monometallic catalysts in hydrocarbon combustion remains under debate [22], whereas for Pt-based systems, Pt^0^ is generally recognized as the primary active site for the catalytic combustion of light hydrocarbons [22].

While specific data on Pd-Pt catalysis for ethylene combustion is unavailable, previous studies have reported enhanced catalytic activity in Pd-Pt bimetallic systems for other organic compounds. In [22], the superior catalytic performance of the Pd-Pt catalyst over monometallic catalysts is explained from the cooperative effect of Pd and Pt active sites via electron transfer effects. It was demonstrated that the synergy of Pd and Pt dual active sites greatly improved the catalytic activity of toluene oxidation. In [23], the improvement in catalytic activity was explained by the fact that Pt facilitates the formation of PdO, which is a better catalyst for methane oxidation. However, this mechanism is less applicable to ethylene, which oxidizes over PdO only at high temperatures (>820 K) [24].

The improved performance of Pd-Pt in this study is likely due to electronic interactions at the bimetallic interface. Regardless of the exact mechanism, our findings demonstrate that Pd-Pt enables effective ethylene and ethanol sensing at relatively low temperatures, making it a promising candidate for low-power, practical sensor applications.

### 3.3. Sensing of High Ethylene Concentrations

**Figure 20:**
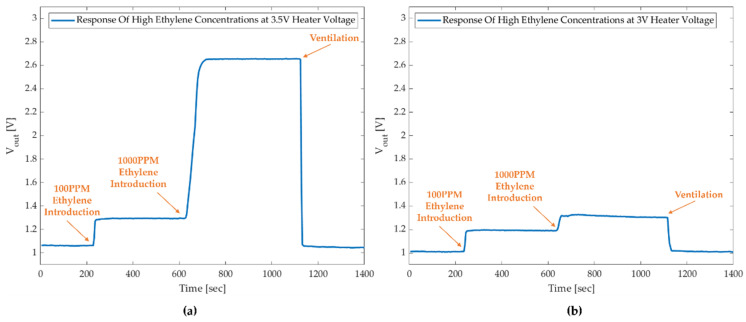
GMOS response to 100 ppm and 1000 ppm of ethylene at (**a**) 3.5 V and (**b**) 3.0 V heater voltage. For a high catalyst temperature (*V_heater_* = 3.5 V), the GMOS signal is proportional to ethylene concentrations. For a lower catalyst temperature (*V_heater_* = 3.5 V), the signal is not proportional to ethylene concentrations.

Along with low-concentration detection for smart agriculture, high ethylene concentration sensing is of great importance in the chemical industry. We investigated the GMOS sensing of 100 and 1000 ppm ethylene using a Pt catalyst. The measurements were performed at heater voltages of 3.0 V and 3.5 V, corresponding to 223 °C and 268 °C catalyst temperatures. The raw GMOS output voltage response is shown for the sequential injection of 100 ppm and 1000 ppm of ethylene into the gas chamber (Figure 20), and a summary of the obtained signals is presented in Table 4.

The results showed that at a catalyst temperature of 268 °C/3.5 V, the GMOS response was proportional to the ethylene concentration (Figure 20a). At a lower catalyst temperature of 223 °C/3.0 V, the response to 100 ppm was adequate; however, the subsequent introduction of 1000 ppm did not result in a proportional response to the increased concentration. This means that the temperature was not high enough to process all the gas molecules coming to the pixel surface, and the active sites of the catalyst surface were clogged. The ethylene concentration effect could also be caused by the inhibition effect of water produced during ethylene combustion, according to the explanation given for high concentration methane combustion in [9]. The apparent self-inhibition effect caused by methane over the Pd-Pt catalyst was found to be associated with the inhibiting effect of water produced during the combustion of methane [9]. For the proper operation of the GMOS sensor in the case of high ethylene concentrations, the heater voltage should be set to 3.5 V or higher.

## 4. Conclusions

Catalyst sensitivity and thermal operating regimes are critical parameters influencing the performance of GMOS gas sensors. In this study, a bimetallic Pd-Pt nanoparticle catalyst was evaluated and compared with monometallic Pd and Pt catalysts for ethylene and ethanol sensing. The Pd-Pt catalyst demonstrated a clear synergistic effect, enabling higher sensitivity and allowing operation in the diffusion-controlled regime at lower temperatures than those required for the individual metals.

Using the same GMOS sensor, a wide ethylene concentration range (5–1000 ppm) was successfully detected. Optimal heating conditions were established for both low and high concentrations. However, accurate detection at high concentrations depends on maintaining sufficiently high catalyst temperatures to ensure the linearity of response.

Activation energy analysis further confirmed the advantage of the Pd-Pt catalyst, which exhibited the lowest activation energy among the three catalysts studied. This suggests more favorable surface reaction kinetics.

Overall, this work demonstrates the potential of Pd-Pt bimetallic catalysts to significantly enhance GMOS sensor performance for combustion-based ethylene detection in agricultural and industrial applications.

## Figures and Tables

**Table 1:** Translation of heater voltage to catalyst temperature [6].

Heater Voltage [V]	1.4	1.6	1.8	2	2.2	2.4	2.5	2.7	2.8	3	3.3	3.5	3.8
Temperature [°C]	89	104	120	136	153	170	179	196	205	223	250	268	294

**Table 2:** Sensitivity counted to 1 ppm (mV/ppm) in the GMOS detection of ethylene and ethanol with different catalysts at two heater voltages. Readout Amplification Gain ≈ 200.

Ethylene Sensitivity [mV/ppm]			
Catalyst	Conc. [ppm]	*V_heater_* = 3.5 V	*V_heater_* = 3.8 V
Pd	100	0.2 ± 0.01	0.4 ± 0.01
Pt	5	2.6 ± 0.08	3.8 ± 0.08
Pd-Pt	5	2.4 ± 0.09	2.4 ± 0.09
**Ethanol Sensitivity [mV/ppm]**			
**Catalyst**	**Conc. [ppm]**	***V_heater_* = 3.5 V**	***V_heater_* = 3.8 V**
Pd	100	0.48 ± 0.01	0.52 ± 0.01
Pt	50	0.52 ± 0.01	0.62 ± 0.01
Pd-Pt	50	0.7 ± 0.02	0.72 ± 0.02

**Table 3:** Summary of activation energies (E_a_), coefficients of determination (R^2^), and associated errors in E_a_ for ethylene and ethanol sensing on Pd, Pt, and Pd–Pd-Pt catalysts.

Ethylene			Ethanol		
Catalyst	E_a_ [KJ/mol]	R^2^	Catalyst	E_a_ [KJ/mol]	R^2^
Pd	50.8 ± 0.5	0.9994	Pd	48.9 ± 0.5	0.9833
Pt	35.5 ± 0.4	0.9985	Pt	39.9 ± 0.2	0.9958
Pd-Pt	29.7 ± 1.1	0.8805	Pd-Pt	31.8 ± 1.1	0.9951

**Table 4:** GMOS signal (V_sig_ [mV]) at 100 ppm and 1000 ppm ethylene concentrations at two catalyst temperatures.

V_heater_ [V]	V_sig_ Obtained for 100 ppm Ethylene [mV]	V_sig_ Obtained for 1000 ppm Ethylene [mV]
3.0	180	130
3.5	235	2360

## Data Availability

The original contributions presented in this study are included in the article/Supplementary Material. Further inquiries can be directed to the corresponding author.

## Supplementary Materials

The following supporting information can be downloaded at: https://www.mdpi.com/article/10.3390/mi16060672/s1, Title S1: Ethylene Gas Concentration Calculations; Title S2: Ethanol Gas Concentration Calculations.
