# Supplementary material for: The Role of Pd-Pt Bimetallic Catalysts in Ethylene Detection by CMOS-MEMS Gas Sensor Dubbed GMOS
Source: Micromachines (Basel). 2025 May 31;16(6):672. doi: 10.3390/mi16060672 (PMC12195325; doi:10.3390/mi16060672)
Supplement: Supplementary file 1 [file micromachines-16-00672-s001.zip › micromachines-3632880-supplementary.pdf]

Supplementary Material

Ethylene Gas Concentration Calculations

To calculate the final concentration of ethylene in the chamber after injecting a known quantity of gas, we apply a simple mass balance under the assumption of ideal mixing and no losses. In this derivation, it is assumed that ethylene gas is introduced into the chamber with a known air volume denoted as V_{ch}. The ethylene is supplied from a gas bottle with a known and constant concentration C_0, expressed in units of ppm. The gas is injected into the chamber at a constant flow rate f for a specific duration t, allowing for a controlled and quantifiable addition of gas to the system.

The volume of ethylene-containing gas ($V_{injected}$) introduced during that time t is given by:

$$V_{injected} = f \cdot t \tag{S1}$$

After injection, the total volume becomes:

$$V_{total} = V_{ch} + f \cdot t \tag{S2}$$

The amount of ethylene introduced is the product of the concentration in the source and the volume delivered:

$$n_{ethylene} = C_0 \cdot f \cdot t \tag{S3}$$

Assuming perfect mixing, the ethylene distributes uniformly throughout the final volume. Therefore, the final ethylene concentration in the chamber can be calculated from Equation (7) described in section 2.3:

$$C_{ch} = \frac{n_{ethylene}}{V_{total}} = \frac{C_0 \cdot f \cdot t}{V_{ch} + f \cdot t} \tag{S4}$$

Ethanol Gas Concentration Calculations

The number of moles of air in the chamber was calculated using the ideal gas law:

$$n_{air} = \frac{P_{ch}V_{ch}}{RT} \tag{S5}$$

where P_{ch} is the atmospheric pressure inside the chamber (1 atm), V_{ch} is the chamber volume (6 L), R is the gas constant (8.314 J/mol·K), and T is temperature (300 K).

The number of air molecules N_{air} is calculated by:

$$N_{air} = n_{air} \cdot N_A \tag{S6}$$

Where N_A is Avogadro's number (6.02*10^{23} 1/mol).

A target concentration of 1 ppm of ethanol corresponds to:

 https://doi.org/10.3390/mi16060672

$$\frac{N_{ethanol}}{N_{air}} = \frac{1}{10^6} \quad \text{(S7)}$$

Thus, the number of moles of ethanol needed is obtained by dividing Equation (S6) by N_A:

$$n_{ethanol,1ppm} = \frac{n_{air}}{10^6}\ [mol] \quad \text{(S8)}$$

To obtain an ethanol concentration of 1 ppm, the volume that needs to be added can be calculated using the equation below:

$$V_{ethanol,1ppm} = \frac{m_{ethanol,1ppm}}{\rho_{ethanol}} = \frac{n_{ethanol,1ppm} M_{w_{ethanol}}}{\rho_{ethanol}}\ [L] \quad \text{(S9)}$$

Where $M_{w_{ethanol}}$ – ethanol molecular mass (46.07 g/mol), $\rho_{ethanol}$ – ethanol density (0.78*10^{-3} gr/L).
By using Equation (S4) and Equation (S7), we can further explicit Equation (S8) to Equation (8) that we presented in section 2.3 in the manuscript:

$$V_{ethanol,1ppm} = \frac{P_{ch} \cdot V_{ch} \cdot M_{w_{ethanol}}}{10^6 \cdot R \cdot T \cdot \rho_{ethanol}} \quad \text{(S10)}$$

Thus, to get X ppm of ethanol, we need to add X times the volume we calculated for 1ppm ($V_{ethanol,1ppm}$).
